# A novel mutation of MSX1 in oligodontia inhibits odontogenesis of dental pulp stem cells via the ERK pathway

**DOI:** 10.1186/s13287-018-0965-3

**Published:** 2018-08-22

**Authors:** Tianyi Xin, Ting Zhang, Qian Li, Tingting Yu, Yunyan Zhu, Ruili Yang, Yanheng Zhou

**Affiliations:** 1 Department of Orthodontics, Peking University School and Hospital of Stomatology, Beijing, 100081 China; 2 National Engineering Laboratory for Digital and Material Technology of Stomatology, Beijing Key Laboratory of Digital Stomatology, Beijing, 100081 China

**Keywords:** MSX1, Odontogenesis, Stem cells, Erk pathway, Tooth agenesis

## Abstract

**Background:**

Tooth agenesis, one of the most common developmental anomalies, can affect the function and esthetics of patients. The aim of the present study was to identify genetic clues for familial tooth agenesis and explore the underlying mechanisms, focusing on the role of human dental pulp stem cells (hDPSCs).

**Methods:**

We applied Sanger sequencing to identify the cause of oligodontia in a Chinese family. DNA transfection and functional analysis in DPSCs was also performed to explore the impact of the identified mutation on this phenotype.

**Results:**

In this study, a novel frameshift mutation, the twenty-nucleotide deletion (c.128_147del20, p.Met43Serfsx125), in exon1 of MSX1 was detected in a Chinese family causing autosomal dominant nonsyndromic oligodontia. The mutation cosegregated with the tooth agenesis phenotype in this family. DPSCs transfected with mutant MSX1 plasmid showed decreased capacity of osteo/odontogenic differentiation with a lower expression level of dentin sialophosphoprotein (DSPP) and bone sialoprotein (BSP) compared with those transfected with control MSX1 plasmid. Mechanically, control MSX1 showed nuclear localization while the mutant MSX1 inhibited its nuclear translocation and localized on the cytoplasm to inhibit ERK phosphorylation. Furthermore, we inhibited the ERK pathway using ERK inhibitor (U0126) treatment in control MSX1-transfected DPSCs which could downregulate mineralized nodule formation and the expression of odontogenic genes.

**Conclusion:**

We demonstrated a novel MSX1 mutation causing familial nonsyndromic oligodontia and mechanically MSX1 regulates odontogenesis through the ERK signaling pathway in human dental pulp stem cells.

**Electronic supplementary material:**

The online version of this article (10.1186/s13287-018-0965-3) contains supplementary material, which is available to authorized users.

## Background

Tooth agenesis is one of the most common developmental anomalies affecting the function and esthetics of patients. Nonsyndromic tooth agenesis can be categorized into three types according to the number of congenitally missing teeth: hypodontia (less than six missing teeth excluding the third molar), oligodontia (six or more missing teeth excluding the third molar), and anodontia (all teeth missing). The prevalence of tooth agenesis of permanent teeth in the general population ranges from 2.2% to 10.1% [1]. Tooth agenesis may contribute to a lot of problems including malocclusion, masticatory dysfunction, speech disorder, and esthetic problems, etc.

Mutations in numerous genes including msh homeobox 1 (MSX1), paired box 9 (PAX9), Wnt family member 10A (WNT10A), axis inhibition protein 2 (AXIN2), ectodysplasin A (EDA), and Wnt family member 10B (WNT10B) have been associated with nonsyndromic oligodontia [2–7]. MSX1 belongs to a family of transcription factors which are expressed at various sites of tissue interactions in overlapping patterns during the development of vertebrates [8]. Mice lacking the MSX1 protein function manifest a cleft secondary palate, a deficiency of alveolar bone, and a failure of tooth development [9]. In humans, MSX1 defects have been reported to cause sporadic or familial nonsyndromic tooth agenesis [10–16]. However, the mechanism of how MSX1 mutation results in tooth agenesis is still not fully understood.

In 2000, Shi et al. first identified dental pulp stem cells (DPSCs), a population of mesenchymal stem cells that possesses the properties of high proliferative potential, the capacity for self-renewal, and multilineage differentiation [17, 18]. The precise and timely regulation of DPSC renewal and differentiation is essential for odontogenesis and reparation of pulp tissue [19, 20].

During tooth formation, tooth morphogenesis is promoted by interactions between epithelial and dental papilla cells through stimulating DPSCs to differentiate into odontoblasts, which in turn form primary dentin. After tooth eruption, odontoblasts which are differentiated from DPSCs in response to injury form reparative dentin. When combined with hydroxyapatite (HA)/tricalcium phosphate (TCP) powder and transplanted into immunocompromised mice, DPSCs generate a dentin-like structure which lines the surfaces of the HA/TCP particles composed of a highly ordered collagenous matrix deposited along the odontoblast-like layer and which expresses dentin sialophosphoprotein (DSPP), the dentin-specific protein [18]. Besides ectopic pulp-dentin complex formation in immunocompromised mice, researchers also observed regeneration of vascularized pulp-like tissue with a layer of newly deposited dentin-like tissue along the canal walls using DPSCs carried by hydrogel and transplanted into the mini-swine root canal space [21].

In the present study, we identified a novel frameshift mutation of MSX1 in a Chinese family which manifested as autosomal dominant nonsyndromic oligodontia, and explored how the mutation in MSX1 affects the function of DPSCs via the extracellular signal-related kinase (ERK) pathway.

## Methods

### Pedigree construction and clinical diagnosis

The proband of the family came to the Department of Orthodontics in Peking University School and Hospital of Stomatology to have orthodontic treatment. Panoramic radiographs confirmed the diagnosis of oligodontia. The pedigree of the family was made by clinical examination and interviews with the available family members. Written informed consent was signed by all participants. This study was approved by the Institutional Ethics Committee of Peking University School and Hospital of Stomatology (PKUSSIRB-2013053).

### Mutation detection

Genomic DNA of all the family members was extracted from peripheral blood using the QIAmp Blood minikit (Qiagen, Venlo, the Netherlands). Mutation screening of MSX1 was performed using polymerase chain reaction (PCR) and direct sequencing. The coding regions of MSX1, including exon-intron boundaries, were amplified by PCR with primers designed using Primer Premier 5.0 software. Once the mutation was detected, the PCR products were purified using 1% agarose gel electrophoresis and cloned into pZeroBack/Blunt Vector (Tiangen, China) to examine the exact site of mutation.

### Construction of MSX1 expression vectors and site-directed mutagenesis

The human MSX1 cDNA with Flag-tag was cloned into pcDNA6.0 expression vector, and pcDNA6.0-MSX1 was obtained, comprising a Flag-tag at the N-terminus of MSX1. The c.128_147del20 mutant was generated using the following two pairs of primers: forward1, 5’-CCCAAGCTTGCCACCATGGACTACAAAG-3’; reverse1, 5’-TTTGGGCTTGGCTGGCGGCTGCCGTGGCCGCGG-3’; forward2, 5’-ACGGCAGCCGCCAGCCAAGCCCAAAGTGTCCCCT-3’; and reverse2, 5’-CGGGATCCCTATGTCAGGTGGTACAT-3’, which deleted twenty nucleotides and made the coding sequence terminate early.

### Cell culture

Human dental pulp stem cells (hDPSCs) were isolated from healthy donors and cultured with complete medium (alpha modification of Eagle’s medium, 15% fetal bovine serum, 100 μg/mL glutamine, 100 μg/mL penicillin, and 100 μg/mL streptomycin) under an environment of 5% CO_2_ at 37 °C. The procedure to acquire human tissues was approved by the Ethics Committee of Peking University School and Hospital of Stomatology (PKUSSIRB-201311103).

### MSX1 knockdown

We purchased MSX1 small interfering RNA (siRNA) oligonucleotides from GenePharma (Suzhou, China). The sequences were: 5’-GCAUUUAGAUCUACACUCUtt-3′ (sense) and 5′- AGAGUGUAGAUCUAAAUGCta-3′ (anti-sense). The sequences for the negative control were 5’-UUCUCCGAACGUGUCACGUtt-3′ (sense) and 5’-ACGUGACACGUUCGGAGAAtt-3′ (antisense). MSX1 siRNA and negative control were transfected into cells with lipofectamine RNAiMAX Reagent (Invitrogen, USA). After 48 h, cells were harvested for analysis or cultured under osteo/odontogenic conditions.

### Transient transfection

We overexpressed wild-type or mutant Flag-MSX1 in DPSCs with lipofectamine LTX (Invitrogen, USA) following the manufacturer’s instructions. After 24 h of culturing with the complete medium or 14 days of culturing under osteo/odontogenic conditions, cells were analyzed by flow cytometry, immunofluorescent staining, western blot, quantitative reverse transcription (RT)-PCR or Alizarin Red S staining.

### Flow cytometry assays

Cultured cells were collected by centrifugation and incubated for 1 h, and the following fluorescently conjugated antibodies were used for cell surface markers: CD29, CD73, CD34, and CD45 (BD Biosciences, USA). The cell proliferation rate was examined using anti-human-ki67 (BD Biosciences, USA). Phycoerythrin (PE)- or fluorescein isothiocyanate (FITC)-Mouse IgG1 (BD Biosciences, USA) were used as isotype controls to set appropriate gates. A BD Accuri C6 flow cytometer platform (BD Biosciences, USA) was used to perform analysis of the stained cells. For each sample, approximately 50,000 cells were analyzed to form the scatter plots.

### Fluorescent immunocytochemistry

Cells were harvested 48 h after transfection of siRNA or 24 h after transfection of plasmids and fixed in 4% paraformaldehyde, followed by permeabilization with 0.1% Triton X-100, and then blocked with 5% bovine serum albumin. The slides were then incubated with anti-MSX1 antibody (1:100; R&D systems, USA) or anti-Flag antibody (1:150; Zhongshanjinqiao, China) for 1 h at room temperature, and mounted with medium containing 4’,6’-diamidino-2-phenylindole (DAPI) to stain the nuclei. The slides were then observed by fluorescence microscopy.

### Western blot analysis

Transfected cells were lysed with RIPA buffer mixed with protease and phosphatase inhibitor cocktail (Thermo Fisher Scientific, Rockford, IL, USA). Total protein (20 μg) was separated by 10% SDS-polyacrylamide gel and transferred onto a polyvinylidene difluoride (PVDF) membrane (Millipore, USA). After being blocked in 5% nonfat milk in Tris-buffered saline containing 0.1% Tween-20 for 1 h at room temperature, the membranes were then incubated with anti-MSX1 (R&D systems, USA), anti-Flag (Zhongshanjinqiao, China), anti-β-actin (Sigma, USA), anti-ERK, and anti-phospho-ERK (Cell Signaling Technology, USA) overnight at 4 °C. They were then treated with horseradish peroxidase-conjugated mouse or rabbit IgG (1:10,000; Zhongshanjinqiao, China) for 1 h at room temperature. After washing with Tris-buffered saline containing 0.1% Tween-20, the membrane was detected by ECL Plus blotting detection reagents (Thermo Fisher Scientific, Rockford, IL, USA) under a western blot detection system (Bio-rad, USA).

### Quantitative real-time polymerase chain reaction

Quantitative RT-PCR was applied to examine the expression of DSPP and bone sialoprotein (BSP). Total RNA of the treated cells was extracted by TRIzol reagent (Invitrogen, USA) and synthesis of cDNA was performed using the SuperScript RT-PCR System (Thermo Fisher Scientific, Rockford, IL, USA) with Platinum Taq High Fidelity (Invitrogen, USA). Quantitative RT-PCR was performed on a Real-Time PCR System (Applied Biosystems 7500, USA) using 2×SYBR Green (Invitrogen Life Technologies, USA). The following primers were used: *GAPDH*, forward 5’-GGAGCGAGATCCCTCCAAAAT-3’ and reverse 5’-GGCTGTTGTCATACTTCTCATGG-3’; *DSPP*, forward 5’-TGGCGATGCAGGTCACAAT-3’ and reverse 5’-CCATTCCCACTAGGACTCCCA-3’; *BSP*, forward 5’-AAAGTGAGAACGGGGAACCT-3’ and reverse 5’-GATGCAAAGCCAGAATGGAT-3’.

### Alizarin Red S staining

The number of calcium nodules formed by hDPSCs after transfection of MSX1 siRNA, wild-type, or mutant MSX1 was analyzed by Alizarin Red S staining. After culture in the osteo/odontogenic medium (complete culture medium containing 10^–7^ M dexamethasone, 0.05 mM ascorbic acid 2-phosphate, and 10 mM β-glycerophosphate) for 14 days, the cells were fixed using 60% isopropanol and stained with 1% Alizarin Red S (Sigma, USA) at room temperature. The cells were then washed with deionized water several times and visualized under a light microscope to analyze the mineralized nodule formation.

### Statistical analysis

Differences between two groups were analyzed using the Student’s *t* test using IBM SPSS Statistics 20 software (IBM Corp., NY, USA) and more than two groups were analyzed by one-way analysis of variance (ANOVA). *P* < 0.05 was considered significant.

## Results

### Clinical diagnosis of oligodontia in a Chinese family

**Fig. 1:**
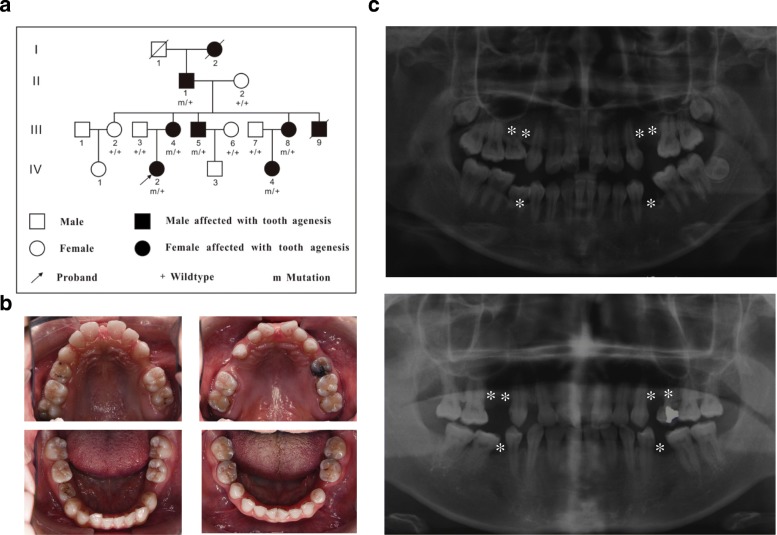
A Chinese family with autosomal dominant nonsyndromic oligodontia. **a** Pedigree of the affected family. Arrow indicates the proband. **b** Intraoral photos of the proband (left) and her mother (right). **c** Panoramics of the proband (top) and her mother (bottom). The proband and her mother lacked six permanent teeth including all maxillary premolars and mandibular second premolars (denoted by asterisks)

The pedigree of the family was made by clinical examination and interviews with the available family members (Fig. 1a). The proband of the family (IV-2) was a systemically healthy 18-year-old female. Clinical and radiographic examinations showed that the proband lacked all maxillary premolars and mandibular second premolars, and also the lower right third molar (Fig. 1b, c). The mother of the proband (III-4) was missing six premolars and all third molars (Fig. 1b, c), while the father of the proband (III-3) had normal dentition. Furthermore, five other family members (II-1, III-5, III-8, and IV-4) manifested similar tooth agenesis according to our clinical and radiographic examination. Another two members who had passed away also had similar oral manifestations according to a description from other family members. All family members denied any medical history of abnormalities in other organs including the sweat glands, hair, skin, nails, or any other systemic disorder, indicating a nonsyndromic tooth agenesis phenotype.

### The identified oligodontia showed mutation of MSX1

**Fig. 2:**
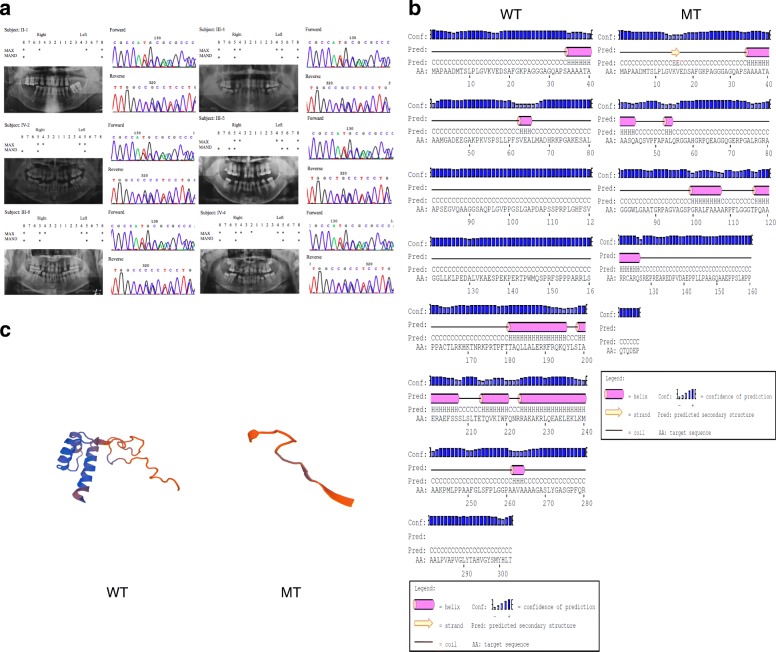
Mutation analysis of the family. **a** Panoramic radiographs and Sanger chromatograms of all the affected members in the family. Schematic analysis shows the position of congenitally missing teeth (denoted by asterisks) in each of the individuals. **b** The predicted secondary structure of wild-type (WT) and mutant (MT) MSX1 protein. The translation of mutant MSX1 terminated earlier than the wild-type. **c** Three-dimensional models of wild-type and mutant MSX1. The mutant MSX1 lost the homeodomain structure to bind DNA

Sanger sequencing analysis showed that the proband of the family carried a twenty-nucleotide deletion (c.128_147del20, p.Met43Serfsx125), which resulted in frameshift and premature translation termination of MSX1 (Fig. 2a). We then performed direct sequencing of MSX1 in all of the available family members and found this frameshift mutation cosegregated with the tooth agenesis phenotype in this family with a complete penetrance (Fig. 2a). The PSIPRED and Swiss model analyses showed that the predicted mutant MSX1 lost the homeodomain structure, which plays a vital role for the function of MSX1 as a transcription factor (Fig. 2b, c).

### DPSC proliferation was decreased when transfected by mutant MSX1

**Fig. 3:**
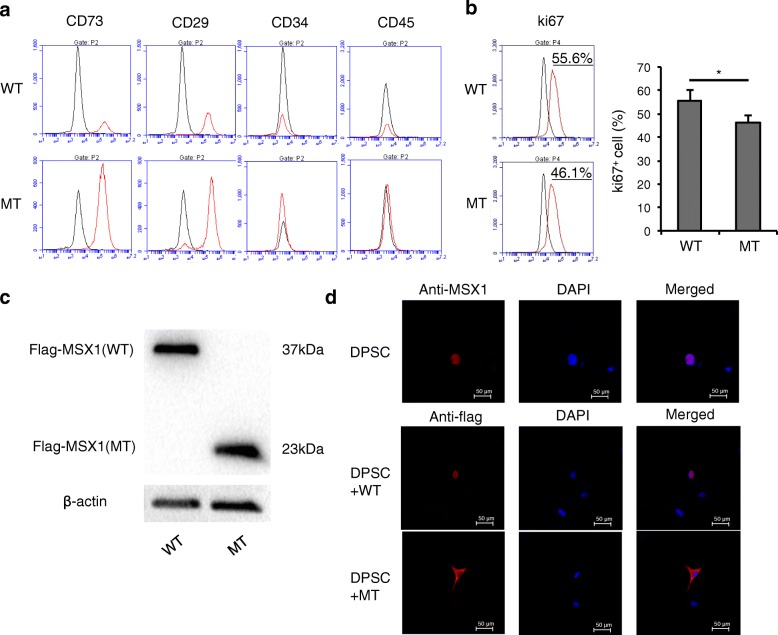
Identification of human dental pulp stem cells (DPSCs) and nuclear localization of MSX1. **a** DPSCs transfected with wild-type (WT) and mutant (MT) MSX1 were positive for CD73 and CD29, and negative for CD34 and CD45. **b** DPSCs transfected with mutant MSX1 showed lower ki67^+^ cell numbers compared with cells transfected with wild-type MSX1. **c** Western blot analysis of cell total protein showed that the molecular weight of mutant MSX1 was remarkably lower than the wild-type. **d** Immunolocalization of intrinsic MSX1 and transfected flag-tagged MSX1 in DPSCs. Blue shows artificial color of nuclei by 4’,6’-diamidino-2-phenylindole (DAPI); red shows anti-flag tag staining. Intrinsic MSX1 localized exclusively in the nucleus. Wild-type MSX1 located exclusively in the nucleus as well, while mutant MSX1 was distributed over the entire cytoplasm. *P < 0.05. Data are expressed as the mean ± SD. Each experiment was repeated three times with *n* ≥ 3 samples per group

Human DPSCs from healthy donors were transfected with wild-type and mutant MSX1. Flow cytometry revealed that DPSCs transfected with wild-type and mutant MSX1 were both positive for mesenchymal stem cell surface markers CD29 and CD73, and negative for hematopoietic stem cell markers CD34 and CD45 (Fig. 3a). However, DPSCs transfected with mutant MSX1 showed a lower ki67^+^ cell number compared with cells transfected with wild-type MSX1, indicating that the proliferation of DPSCs was decreased after mutant MSX1 transfection compared with the control MSX1 transfection (Fig. 3b).

### Mutant MSX1 inhibited nuclear translocation of MSX1 protein

To explore the underlying mechanism, we over expressed wild-type and mutant MSX1 with a Flag-tag in human dental pulp stem cells in vitro. Western blot results showed that both wild-type and mutant MSX1 were expressed, while the molecular weight of mutant MSX1 was remarkably lower than wild-type (Fig. 3c), consistent with the frameshift mutation. To detect intrinsic MSX1 expression in DPSCs, we performed immunofluorescence staining and the results showed that intrinsic MSX1 localized exclusively in the nucleus (Fig. 3d). Interestingly, immunofluorescence staining showed that the transfected wild-type MSX1 located exclusively in the nucleus as well, whereas the mutant MSX1 protein was localized in the entire cytoplasm (Fig. 3d). The result implied that the frameshift mutation of MSX1 inhibited nuclear translocation of this transcription factor.

### MSX1 knockdown inhibited osteo/odontogenic differentiation of hDPSCs

**Fig. 4:**
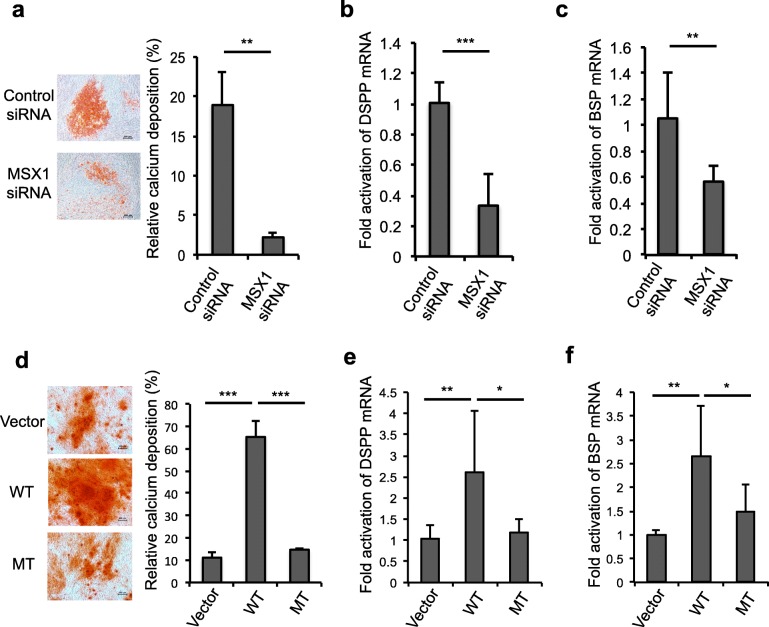
Effects of MSX1 on osteo/odontogenic differentiation of DPSCs. **a** MSX1 knockdown significantly decreased the capacity of osteo/odontogenic differentiation of hDPSCs compared with control. **b**, **c** Quantitative RT-PCR showed that dentin sialophosphoprotein (DSPP) and bone sialoprotein (BSP) was significantly downregulated after MSX1 knockdown compared with control. **d** Calcium nodule deposition of DPSCs after transfection was examined by Alizarin Red S staining. Mutant (MT) MSX1 showed decreased capacity of osteo/odontogenic differentiation compared with those ones transfected with wild-type (WT) MSX1. **e**, **f** Quantitative RT-PCR showed that DSPP and BSP was upregulated when hDPSCs were transfected with wild-type MSX1, while mutant MSX1 transfection showed lower expression levels compared with the wild type. *P < 0.05, **P < 0.01, ***P < 0.001. Data are expressed as the mean ± SD. Each experiment was repeated three times with *n* ≥ 3 samples per group. siRNA small interfering RNA

To investigate the effect of MSX1 knockdown on osteo/odontogenic differentiation of hDPSCs, cells were cultured under osteo/odontogenic conditions after MSX1 siRNA transfection. After 14 days, calcium nodule deposition was examined by Alizarin Red S staining. The results showed that MSX1 knockdown significantly decreased the capacity of osteo/odontogenic differentiation of hDPSCs compared with the control (Fig. 4a). Furthermore, quantitative RT-PCR showed that both DSPP and BSP expression was downregulated after MSX1 knockdown (Fig. 4b, c).

### Mutation of MSX1 inhibited osteo/odontogenic differentiation of hDPSCs

To investigate whether the mutant MSX1 affects the function of hDPSCs, hDPSCs were cultured with osteo/odontogenesis induction medium for 14 days after transfection of wild-type and mutant MSX1, respectively, and calcium nodule deposition was examined by Alizarin Red S staining. The results revealed that mutant MSX1 showed a decreased capacity for osteo/odontogenic differentiation compared with those transfected with wild-type MSX1 (Fig. 4d). Both DSPP and BSP were markedly upregulated when hDPSCs were transfected with wild-type MSX1 in comparison with the vector, while mutant MSX1 transfection showed lower expression levels compared with those transfected with wild-type MSX1 (Fig. 4e, f).

### MSX1 regulates odontogenesis of hDPSCs through the ERK pathway

**Fig. 5:**
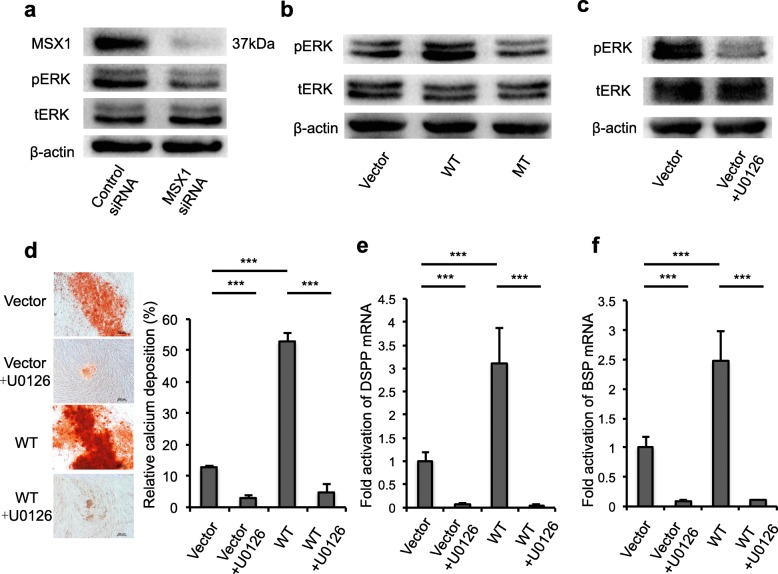
MSX1 regulated odontogenesis of DPSCs through the ERK pathway. **a** MSX1 knockdown significantly decreased ERK phosphorylation (pERK) compared with control. **b** DPSCs transfected with mutant (MT) MSX1 expressed lower levels of phosphorylated ERK in comparison with wild-type (WT) MSX1. **c**, **d** Alizarin Red S staining showed that U0126 decreased odontogenesis of DPSCs compared with vector. The increased mineralized nodule formation induced by MSX1 transfection was blocked after the inhibition of the ERK pathway. **e**, **f** Quantitative RT-PCR showed that U0126 decreased dentin sialophosphoprotein (DSPP) and bone sialoprotein (BSP) expression compared with vector. The increased expression of DSPP and BSP induced by wild-type MSX1 transfection was attenuated after inhibition of the ERK signaling pathway. ***P < 0.001. Data are expressed as the mean ± SD. Each experiment was repeated three times with *n* ≥ 3 samples per group. siRNA small interfering RNA

**Fig. 6:**
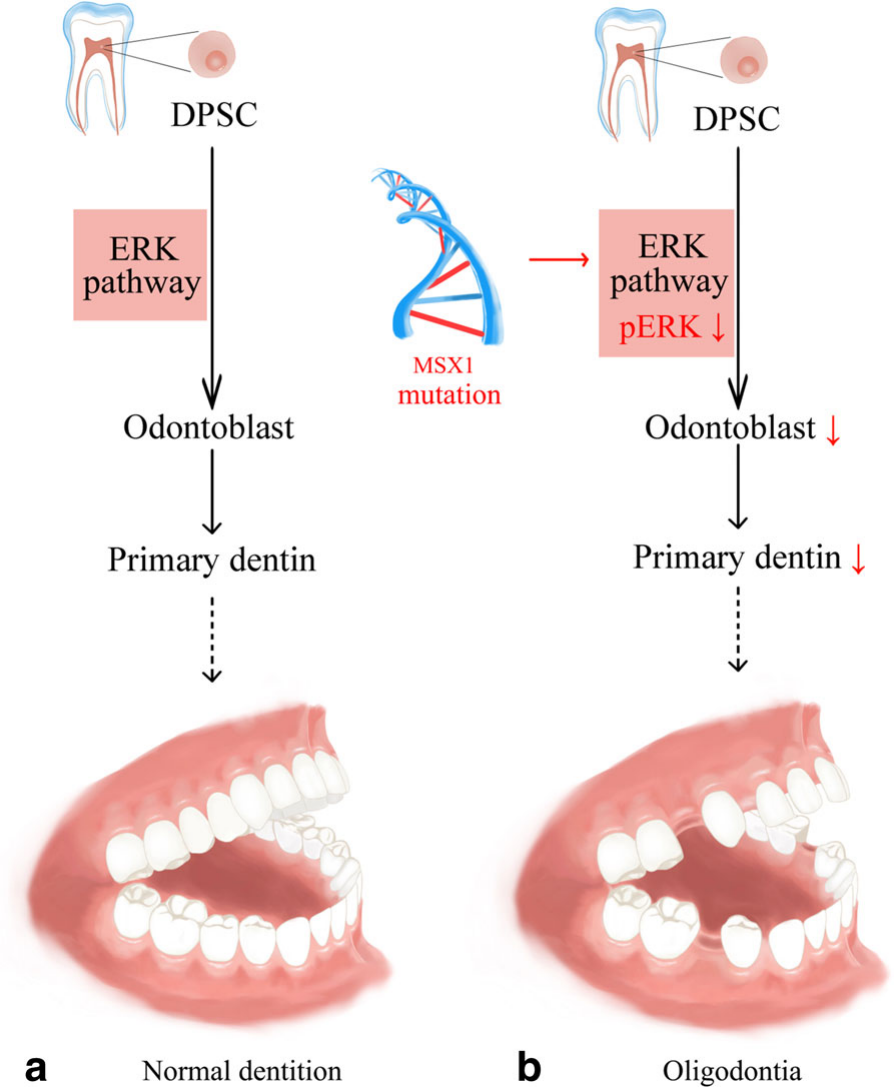
Schematic showing that MSX1 mutation inhibits the odontogenic function of human dental pulp stem cells (DPSCs) via the ERK signaling pathway, thus leading to the oligodontia phenotype in patients. **a** Normal dentition. **b** Oligodontia

It has been reported that the ERK pathway plays an important role in odontogenic differentiation of hDPSCs [22]. Western blot results showed that MSX1 knockdown significantly downregulated ERK phosphorylation in hDPSCs compared with control (Fig. 5a). Wild-type MSX1 transfection significantly increased ERK phosphorylation compared with the vector. However, DPSCs transfected with mutant MSX1 expressed lower levels of phosphorylated ERK in comparison with wild-type MSX1 (Fig. 5b). To further investigate the role of ERK signaling in odontogenic differentiation, DPSCs were cultured under odontogenic differentiation treated with 10 μmol/L U0126 (ERK inhibitor) after transfection of vector or wild-type MSX1. Alizarin Red S staining showed that U0126 decreased odontogenesis of DPSCs compared with vector. Also, the increased mineralized nodule formation induced by wild-type MSX1 transfection was blocked after the inhibition of the ERK pathway (Fig. 5c, d). Quantitative RT-PCR showed that U0126 decreased DSPP and BSP expression compared with vector. The increased expression of DSPP and BSP induced by wild-type MSX1 transfection also was attenuated after the inhibition of the ERK signaling pathway (Fig. 5e, f). Furthermore, we treated hDPSCs with U0126 first, and then transfected wild-type and mutant MSX1 into the cells. The confocal microscopic images showed that nuclear translocation of either wild-type MSX1 or mutant MSX1 was affected, indicating that activation of ERK1/2 is not required for nuclear translocation/activity of MSX1 (Additional file 1: Figure S1a). Western blot results showed that MSX1 levels in U0126-treated cells were comparable to controls (Additional file 1: Figure S1b). Together, these results confirmed that the newly found frameshift mutation of MSX1 inhibited the odontogenic function of hDPSC via *the* ERK signaling pathway, thus leading to the oligodontia phenotype in the patients. (Fig. 6).

## Discussion

We identified a novel frameshift mutation (c.128_147del20, p.Met43Serfsx125) of MSX1 in a Chinese family which manifested as autosomal dominant nonsyndromic oligodontia. The mutation cosegregated with the oligodontia phenotype in this family with complete penetrance. Previous studies have identified more than ten mutations related to nonsyndromic tooth agenesis in MSX1 [10–16, 23–26]. MSX1-deficient mice showed a series of abnormalities in skeletal and tooth development, including the absence of incisor tooth buds and developmental retardation of molar tooth buds [9]. MSX1 function is required in the dental mesenchyme for tooth bud development, especially the progression of molar tooth development beyond the bud stage [27].

Here, in the present study, we first confirmed intrinsic MSX1 expression and investigated its role in odontogenic differentiation of hDPSCs. The results indicated that MSX1 is essential during odontogenic differentiation of hDPSCs. To further analyze how the novel mutation of MSX1 caused tooth agenesis, we constructed wild-type MSX1 expression vectors and performed site-directed mutagenesis to obtain the mutant MSX1 expression vector. Both of the vectors could be expressed in vitro, but wild-type MSX1 showed nuclear localization while the mutant MSX1 localized exclusively in the cytoplasm. It has been reported that nuclear localization is mediated by the homeodomain of MSX1. MSX1 belongs to homeoproteins which are transcription factors that regulate cell proliferation and differentiation. Homeodomain is a highly conserved polypeptide which enables MSX1 to interact with target DNA sequences and several other proteins [28, 29]. The c.128_147del20 mutation made the translation terminate before the homeodomain of MSX1, thus explaining the translocation abnormality of the mutant.

Human DPSCs are capable of both self-renewal and multilineage differentiation, and possess the ability to regenerate dentin pulp-like complex [18]. It has been reported that MSX1 is expressed at higher levels in hDPSCs than in bone marrow-derived mesenchymal stem cells and fibroblasts [30]. We repeated the proliferation experiments independently and found that the proliferation rate of mutant MSX1 transfection DPSCs was significantly lower that wild-type MSX1 transfection DPSCs. This result indicated that MSX1 plays a role in hDPSC proliferation. MSX1 knockdown in DPSCs resulted in a decreased capacity for odontogenic differentiation compared with the control. Wild-type MSX1 transfection increased calcium nodule formation and DSPP and BSP expression compared with vector. DPSCs transfected with mutant MSX1 plasmid showed a decreased capacity for odontogenic differentiation with a lower expression level of DSPP and BSP compared with those transfected with wild-type MSX1 plasmid. These results demonstrated that MSX1 plays an important role in the odontogenic differentiation of hDPSCs. To the best of our knowledge, the present study is the first to apply a mechanistic study of tooth agenesis-related mutation based on hDPSCs. These results indicated that the mutation of MSX1 we identified in the family with autosomal dominant oligodontia may downregulate the ability of hard tissue differentiation by inhibiting the function of hDPSCs, thus leading to the failure of tooth bud formation.

Furthermore, we found that ERK phosphorylation was significantly decreased after MSX1 knockdown in hDPSCs compared with the control. Wild-type MSX1 transfection increased ERK phosphorylation when compared with vector, while mutant MSX1 transfection decreased ERK phosphorylation in comparison with the those transfected with wild-type MSX1. It has been reported that the ERK pathway plays an important role in odontogenic differentiation of hDPSCs. Expression of odontogenic genes such as DSPP and BSP in hDPSCs can be partially abolished by an ERK inhibitor [22]. When we add U0126 to hDPSCs after transfection of wild-type MSX1, the upregulation of odontogenic genes and the promoted osteo/odontogenic differentiation of hDPSCs were inhibited. These results reveal, for the first time, that MSX1 regulates odontogenesis of hDPSCs via the ERK pathway. In developing tooth and cranial sutures, MSX1 expression has been reported to be regulated by fibroblast growth factors (FGFs) [31], while FGF is known to induce ERK activation. Our results revealed that MSX1 might play an important role in FGF-induced ERK activation. However, how MSX1 specifically activates ERK1/2, and the factors affecting MSX1 transcriptional activity during ERK1/2 phosphorylation, may need further investigation. These underlying mechanisms may provide insights into novel therapeutic strategies for patients with tooth agenesis caused by MSX1 mutation. Improvement of hDPSC function through ERK pathway regulation may be considered a treatment for patients with a tooth agenesis-related genetic defect in the future.

## Conclusion

In conclusion, the present study identified a novel frameshift mutation of MSX1 associated with autosomal dominant nonsyndromic oligodontia in a Chinese family. Subsequent in-vitro studies proved that this newly found MSX1 mutation inhibited the odontogenic function of hDPSCs via the ERK signaling pathway (Fig. 6). Understanding these mechanisms will provide insights into novel therapeutic strategies for patients with tooth agenesis caused by MSX1 mutation in the future.

## Additional file

Additional file 1: **Figure S1.** ERK activity is not required for MSX1 expression. (A) Immunolocalization of flag-tagged MSX1 protein in U0126-treated DPSCs. Wild-type MSX1 located exclusively in the nucleus, while mutant MSX1 was distributed over the entire cytoplasm. (B) U0126-treated DPSCs showed comparable levels of MSX1 expression as control. WT wild-type MSX1 transfection, MT mutant MSX1 transfection, Control no transfection, U0126 U0126 added. (PDF 2680 kb)

## Abbreviations

AXIN2: Axis inhibition protein 2
BSP: Bone sialoprotein
DAPI: 4′,6′-Diamidino-2-phenylindole
DPSC: Dental pulp stem cell
DSPP: Dentin sialophosphoprotein
EDA: Ectodysplasin A
ERK: Extracellular signal-related kinase
FGF: Fibroblast growth factor
HA: Hydroxyapatite
hDPSC: Human dental pulp stem cell
MSX1: Msh homeobox 1
PAX9: Paired box 9
PCR: Polymerase chain reaction
siRNA: Small interfering RNA
TCP: Tricalcium phosphate
WNT10A: Wnt family member 10A
WNT10B: Wnt family member 10B
