# Supplementary material for: A novel mutation of MSX1 in oligodontia inhibits odontogenesis of dental pulp stem cells via the ERK pathway
Source: Stem Cell Res Ther. 2018 Aug 22;9:221. doi: 10.1186/s13287-018-0965-3 (PMC6106924; doi:10.1186/s13287-018-0965-3)
Supplement: Supplementary file 1 — Figure S1. ERK activity is not required for MSX1 expression. (A) Immunolocalization of flag-tagged MSX1 protein in U0126-treated DPSCs. Wild-type MSX1 located exclusively in the nucleus, while mutant MSX1 was distributed over the entire cytoplasm. (B) U0126-treated DPSCs showed comparable levels of MSX1 expression as control. WT wild-type MSX1 transfection, MT mutant MSX1 transfection, Control no transfection, U0126 U0126 added. (PDF 2680 kb) [file 13287_2018_965_MOESM1_ESM.pdf]

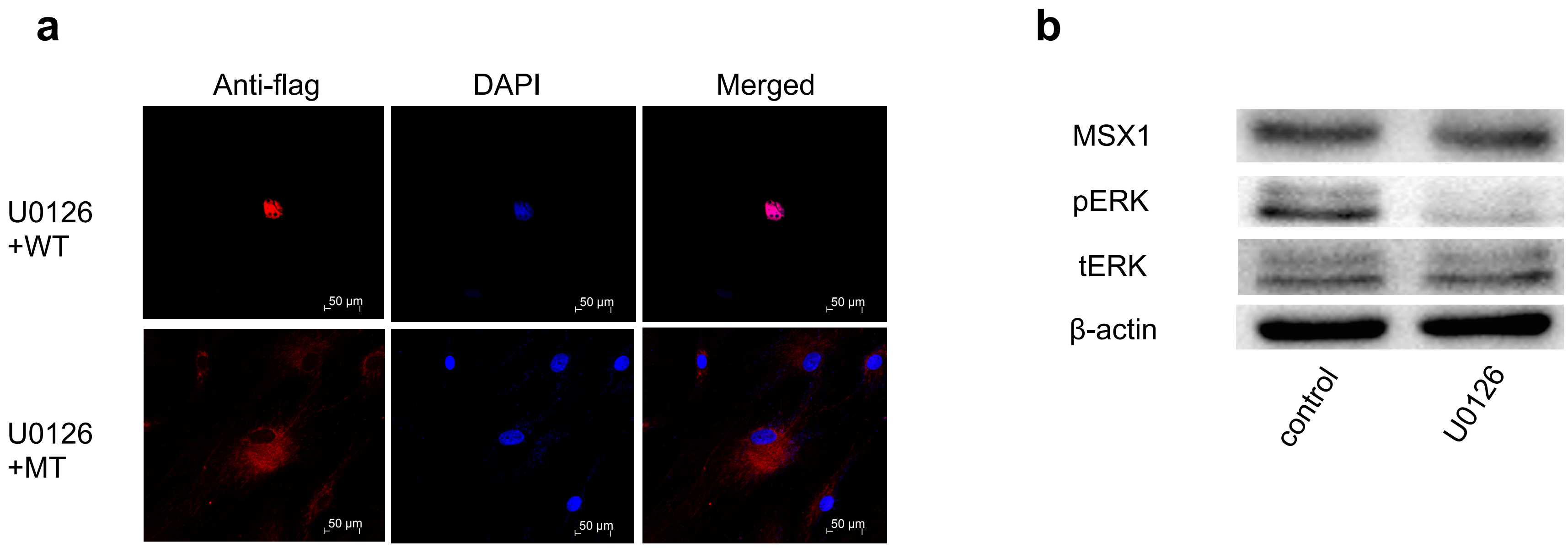

Figure S1. ERK activity is not required for MSX1 expression. (a) Immunolocalization of flag-tagged MSX1 protein in U0126-treated DPSCs. Wild-type MSX1 located exclusively in the nucleus, while mutant MSX1 was distributed over the entire cytoplasm. **(b)** U0126-treated DPSCs showed comparable levels of MSX1 expression as control. WT: wild-type MSX1 transfection, MT: mutant MSX1 transfection, Control: no transfection, U0126: U0126 added
